# Development and Characterization of A Novel Prox1-EGFP Lymphatic and Schlemm’s Canal Reporter Rat

**DOI:** 10.1038/s41598-017-06031-3

**Published:** 2017-07-17

**Authors:** Eunson Jung, Daniel Gardner, Dongwon Choi, Eunkyung Park, Young Jin Seong, Sara Yang, Jorge Castorena-Gonzalez, Antoine Louveau, Zhao Zhou, Gene K. Lee, David P. Perrault, Sunju Lee, Maxwell Johnson, George Daghlian, Maria Lee, Yeo Jin Hong, Yukinari Kato, Jonathan Kipnis, Michael J. Davis, Alex K. Wong, Young-Kwon Hong

**Affiliations:** 1 Division of Plastic and Reconstructive Surgery, Department of Surgery, Keck School of Medicine, University of Southern California, Los Angeles, California USA; 2 Department of Biochemistry and Molecular Medicine, Norris Comprehensive Cancer Center, Keck School of Medicine, University of Southern California, Los Angeles, California USA; 3 Department of Medical Pharmacology and Physiology, University of Missouri, Columbia, Missouri USA; 4 Center for Brain Immunology and Glia, Department of Neuroscience, University of Virginia School of Medicine, Charlottesville, Virginia USA; 5 Department of Regional Innovation, Tohoku University Graduate School of Medicine, Sendai, Japan

## Abstract

The lymphatic system plays a key role in tissue fluid homeostasis, immune cell trafficking, and fat absorption. We previously reported a bacterial artificial chromosome (BAC)-based lymphatic reporter mouse, where EGFP is expressed under the regulation of the Prox1 promoter. This reporter line has been widely used to conveniently visualize lymphatic vessels and other Prox1-expressing tissues such as Schlemm’s canal. However, mice have a number of experimental limitations due to small body size. By comparison, laboratory rats are larger in size and more closely model the metabolic, physiological, and surgical aspects of humans. Here, we report development of a novel lymphatic reporter rat using the mouse Prox1-EGFP BAC. Despite the species mismatch, the mouse Prox1-EGFP BAC enabled a reliable expression of EGFP in Prox1-expressing cells of the transgenic rats and allowed a convenient visualization of all lymphatic vessels, including those in the central nervous system, and Schlemm’s canal. To demonstrate the utility of this new reporter rat, we studied the contractile properties and valvular functions of mesenteric lymphatics, developed a surgical model for vascularized lymph node transplantation, and confirmed Prox1 expression in venous valves. Together, Prox1-EGFP rat model will contribute to the advancement of lymphatic research as a valuable experimental resource.

## Introduction

In mammals, the blood and the lymphatic systems are necessary for proper circulation. These two vasculatures are anatomically similar and complementary in function, whereby the blood vessels carry oxygen and nutrients to tissues, while the lymphatic vessels transport fluid and macromolecules back to the circulation^1^. Despite the significant role that the lymphatic system plays in interstitial fluid drainage, dietary lipid transport, and immune responses, the lymphatic vasculature has been considerably understudied for many decades. Recent progress in lymphatic research, such as the discovery of lymphatic-specific molecular markers^2, 3^, lineage tracing of Prox1-expressing lymphatics^4^, isolation and culturing of lymphatic endothelial cells^5–7^, and the development of various lymphatic-specific mouse models^4, 8–14^, have contributed significantly in illuminating the important role of the lymphatic system in maintaining and promoting human health. The importance of the lymphatic system and the need for an improved mammalian lymphatic model for research are further emphasized by the various disease processes associated with dysfunctional lymphatics including chronic lymphedema, obesity-associated lymphatic dysfunction and chronic inflammation^15^.

We and others have recently reported a Prox1-EGFP lymphatic reporter mouse model. This reporter mouse has been useful for studying many aspects of the lymphatic system^4, 8–13, 16^. However, inherent limitations of mice as an experimental animal model renders it difficult to recapitulate the important role of the lymphatic system as seen in the metabolic, physiologic, and anatomic features of human biology, thus necessitating the development of alternative lymphatic reporter animals that more closely model human biology. We report the successful generation of a transgenic rat model using the engineered mouse Prox1-EGFP BAC that was previously used to create the Prox1-EGFP mouse model^9, 17^. This new lymphatic reporter rat not only enables convenient structural and functional analyses of lymphatic vessels and other Prox1-expressing tissues such as venous valves, but also provides an optimal model to study surgical physiology, such as vascularized lymph node transfer.

## Results and Discussion

To generate a Prox1-EGFP reporter rat model, we initially asked whether the mouse Prox1-EGFP BAC clone, which was previously used to create the Prox1-EGFP mouse model^9, 17^, would be functional in rat tissues. For this purpose, we surveyed the mouse genomic sequences contained in the BAC clone, and found that the genomic sequences were highly conserved in other species, including rat, at their corresponding genomic loci (Supplemental Fig. 1A). In addition, the mouse BAC clone clearly expressed EGFP when transfected in non-mouse cells, such as SW620 (human colon carcinoma)^18^, CHO (Chinese hamster ovary cells)^19^, and L6 (rat myoblasts)^20^ (Supplemental Fig. 1B). These primary data encouraged us to use the mouse BAC clone for generation of Prox1-EGFP reporter rat. Pronuclear microinjection yielded two pups that were positive for the presence of the Prox1-EGFP BAC in their genomes based on PCR-based genotyping. Only one of them expressed clear EGFP signals in its tissues, establishing a founder Prox1-EGFP reporter rat. Prox1-EGFP rats were otherwise healthy and fertile without any detectable abnormalities or diseases, and maintained on normal diet and water ad libitum under standard conditions.

**Figure 1:**
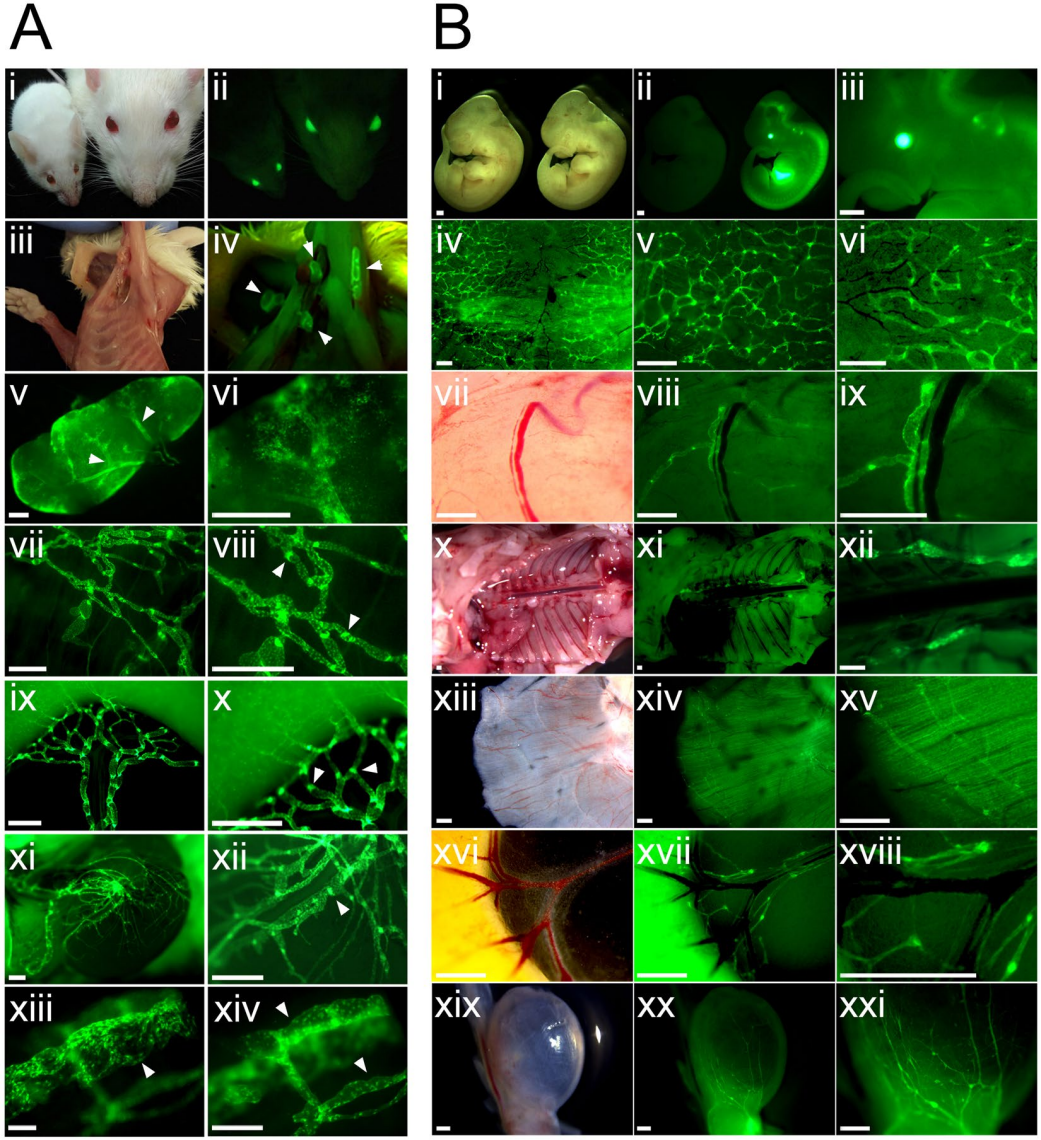
Characterization of the reporter expression in lymphatic vessels of Prox1-EGFP rat. (**A**) EGFP expression patterns in adults: (i,ii) Bright light and fluorescence headshots of Prox1-EGFP mouse (left) and Prox1-EGFP rat (right) highlight the size difference between the two rodents and the strong reporter expression in their lenses. (iii–iv) Cervical and axillary lymph nodes (arrowheads), which are difficult to locate under bright light without a contrasting dye, could be easily identifiable under fluorescence in Prox1-EGFP rats. (v–vi) Fluorescent images showing lymphatic vessels in the capsular and subcapsular area of an adult lymph node. (vii–xvi) Lymphatic vessels and their luminal valves (arrowheads) are clearly visible in the bladder (vii–viii), mesentery (ix–x), and testicle (xi–xii). Schlemm’s canal (arrowhead, xiii) and limbal lymphatic vessels (arrowhead, xiv) of the same eye imaged at different focuses show strong EGFP expression. Scale bars: 500 ${\rm{\mu }}$m (v–xii), 100 µm (xiii–xiv). (**B**) EGFP expression patterns in embryos: (i–iii) Compared to wild type embryo (left), Prox1-EGFP rat embryo (right) shows a strong EGFP expression in the lens, central nervous system, and liver. (iv–vi) Dermal capillary lymphatic network could be easily visualized in the back skin of embryo (E17.5). Panels (v) and (vi) are enlarged images of (iv). (vii–xxi) Bright field and fluorescence images showing lymphatic vessels in the subcutaneous area (vii–ix), thoracic cavity (x–xii), diaphragm (xiii–xv), mesentery (xvi-xviii), and bladder (xix–xxi). Scale bars: 500 µm.

Similar to Prox1-EGFP mice^9^, Prox1-EGFP rats exhibited intense EGFP signals in their eyes under ultraviolet light (Fig. 1A i,ii), as the eye lens strongly express Prox1^21^. Lymph nodes, which are often not readily discernable from surrounding tissues, were also easily localized due to strong EGFP expression (Fig. 1A iii,iv). The perinodal and intranodal lymphatic vessels were also clearly visible in dissected lymph nodes (Fig. 1A v,vi). Similar to Prox1-EGFP mice^9^, Prox1-EGFP rats displayed strong EGFP signals in all lymphatic vessels in the mesentery, skin, and testis (Fig. 1A vii–xii). Moreover, consistent with previous reports showing Prox1 expression in the luminal valves of lymphatic vessels^22, 23^, we found a high expression of EGFP in valvular lymphatic endothelial cells (LECs) (Fig. 1A vii–xii). In addition, distinct structures of Schlemm’s canal and limbal lymphatics were clearly visible with EGFP signals as well (Fig. 1A xiii,xiv).

We next examined the EGFP expression pattern in developing rat embryos. Consistent with the EGFP reporter expression pattern in Prox1-EGFP adult rats, transgenic embryos also revealed a strong EGFP signal in their eyes, liver, and central nervous system (Fig. 1B i–iii), distinguishing the transgenic embryos from their wild type littermates. Moreover, developing lymphatic vessels were conveniently detectable in the back skin, thoracic cavity, diaphragm, intestine, and bladder (Fig. 1B iv–xxi).

**Figure 2:**
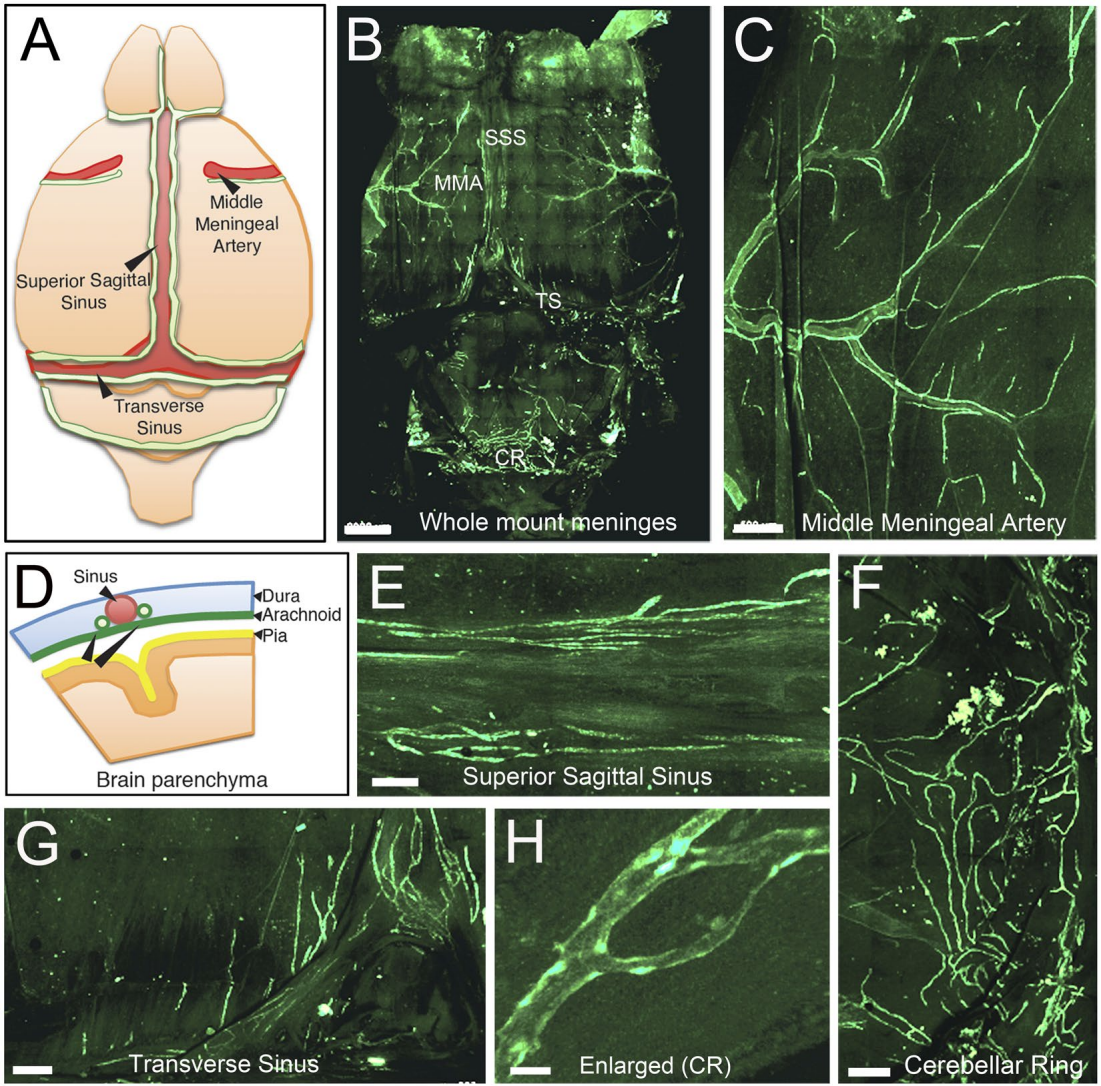
Visualization of meningeal lymphatic vessels in Prox1-EGFP rat. Meninges from Prox1-EGFP rats were dissected and imaged as a whole mount. (**A**) Simplified diagram showing the lymphatics (green) positioned along the veins (superior sagittal and transverse sinuses) and artery (middle meningeal artery) of the rodent meninges. (**B**) Overview of whole mount meninges. *SSS*: Superior Sagittal Sinus, *TS*: Transverse Sinus, *MMA*: Middle Meningeal Artery, *CR*: Cerebellar Ring. (**C**) Lymphatic vessels were easily detected in the area of the middle meningeal artery. (**D**) Scheme of the meninges anatomy. The pia is localized on top of the brain parenchyma, the dura mater lines the skull, and the arachnoid matter lies between the pia and dura mater. The meningeal lymphatics are localized within the dura matter along the sinuses. Lymphatics were also clearly detectable in superior sagittal sinus (**E**), cerebellar ring (**F**), and transverse sinus (**G**). (**H**) Lymphatics in the cerebellar ring are enlarged in panel (**F**). Scale bars: 2 mm (**B**), 500 µm (**C,F,G**), 300 µm (**E**), 30 µm (**H**).

In light of the recent discovery of meningeal lymphatic vasculature in mice^24–26^, we investigated for its presence in our Prox1-EGFP rats. Detailed analyses of the brain of Prox1-EGFP rats confirmed the meningeal lymphatics running next to the middle meningeal artery, superior sagittal sinus, transverse sinuses, and cranial to the cisterna magna (Fig. 2). The meningeal lymphatic vasculature in rats appeared more developed and complex compared to that in mice^24–26^. However, more studies will be needed to systematically elucidate the potential species-specific complexity of the meningeal lymphatic vasculature.

**Figure 3:**
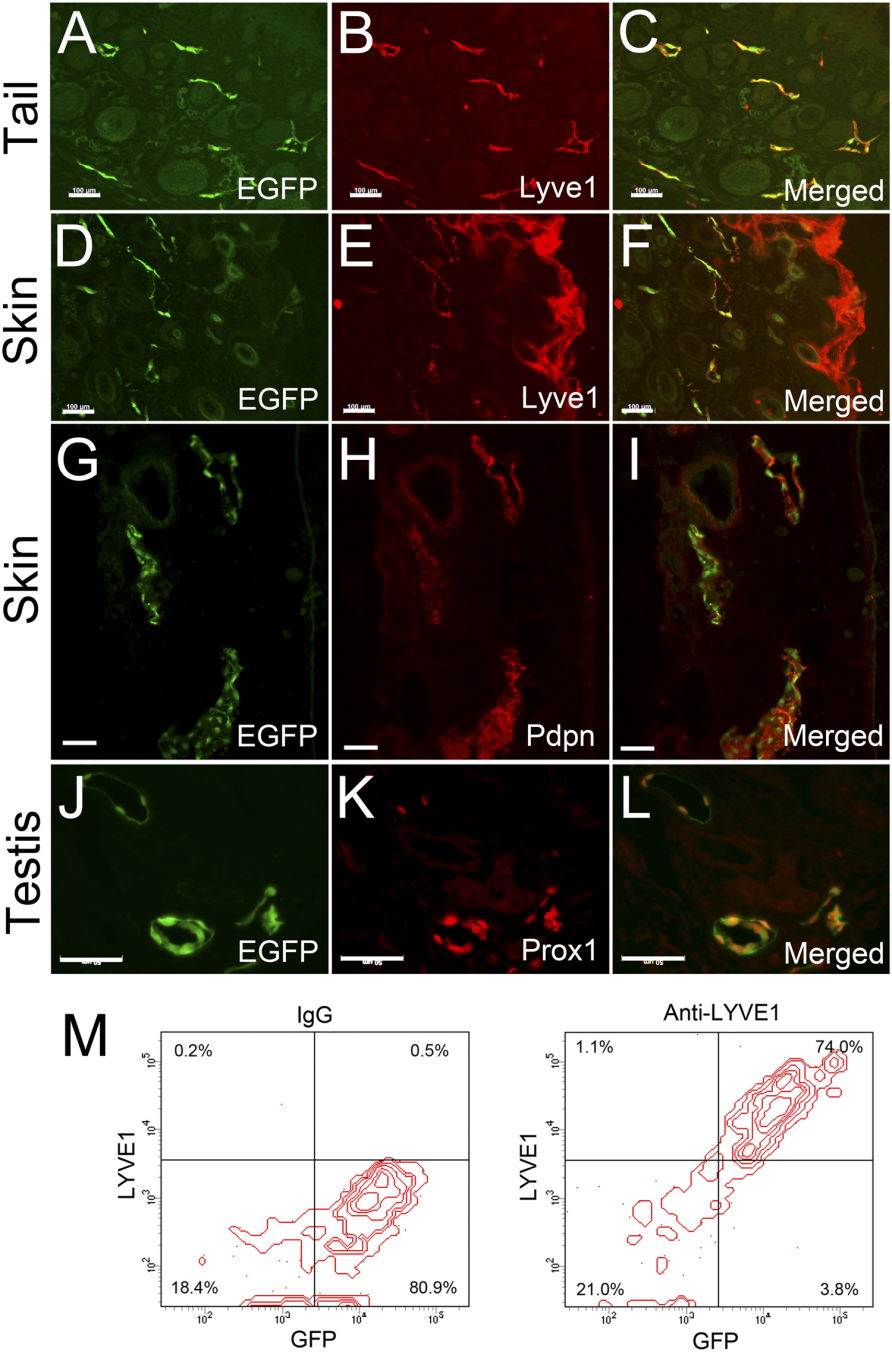
Expression of lymphatic markers in the EGFP-positive vessels of Prox1-EGFP rat. Frozen sections from the tail (**A–C**), skin (**D–I**), and testis (**J–L**) were stained for lymphatic markers, Lyve1, Pdpn, and Prox1 to confirm the expression of lymphatic signature genes in EGFP-positive vessels. Scale bars: 100 μm (**A–I**), 50 μm (**J–L**). (**M**) Flow cytometry analyses showing a predominant Lyve1 expression in primary EGFP-positive cells (LECs) freshly isolated from lymph nodes of Prox1-EGFP rats. Cells were incubated with normal IgG control (left) or anti-Lyve1 antibody (right).

We next set out to confirm the expression of lymphatic markers in the EGFP-positive lymphatic vessels of Prox1-EGFP rat. Frozen sections were prepared from the tail, skin, and testis of Prox1-EGFP adult rats and underwent immunofluorescence analyses for Lyve1, Pdpn and Prox1. Indeed, all tested EGFP-positive vessels were found to clearly express these lymphatic markers (Fig. 3A–L), verifying the lymphatic identity of the EGFP-positive vessels. Moreover, we isolated primary LECs from lymph nodes of Prox1-EGFP rats and evaluated the percentage of cells that express either or both of EGFP and Lyve1. Indeed, while 95.1% of total EGFP-positive cells (presumably LECs) from lymph nodes were found to express Lyve1, 98.5% of Lyve1-positive cells were also positive for EGFP (Fig. 3M). Together, most of EGFP-positive are Lyve1-positive LECs in lymph nodes of Prox1-EGFP rats.

**Figure 4:**
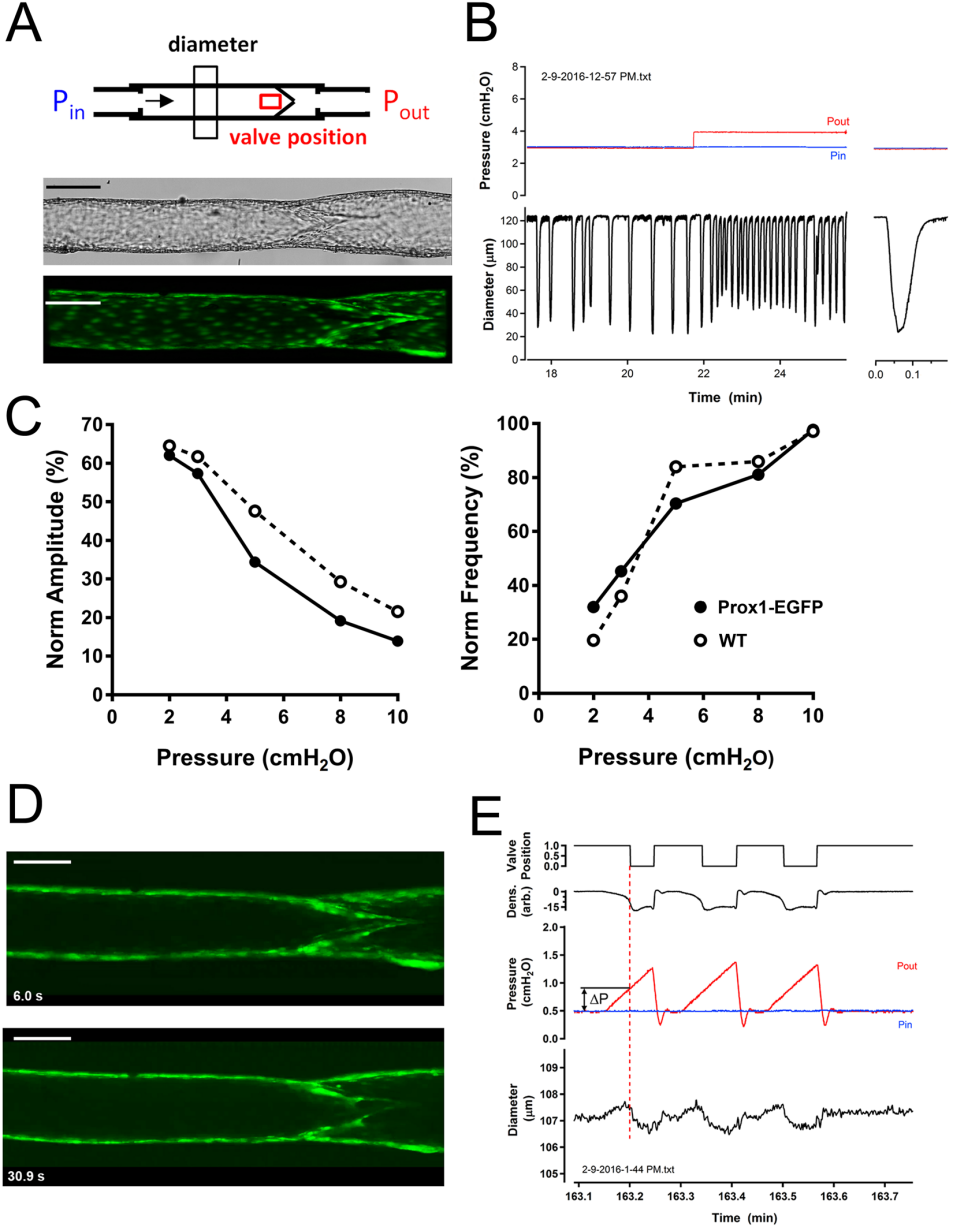
Functional analyses of the mesenteric lymphatics in Prox1-EGFP rat. (**A**) Diagram of an *ex vivo* study system of Prox1-EGFP lymphatic vessels. P_in_ and P_out_ refer to the input and output pressures, respectively, of the two cannulating pipettes. Arrow indicates the normal direction of flow. Windows for diameter tracking (by edge detection) and valve position (by densitometry) are shown by black and red boxes, respectively. Top image is a bright field image of an *ex vivo* vessel; bottom image is a confocal image (maximum intensity projection) of the same vessel. (**B**) Example trace showing spontaneous contractions of a lymphatic vessel when P_in_ and P_out_ were equal and after P_out_ was slightly elevated. Traces were recorded under fluorescence illumination by edge detection of the outer edge of the LEC layer. The response to P_out_ elevation is typical of wild-type vessels (see text). (**C**) Plots of changes in amplitude and frequency as functions of pressure, with both variables averaged over multiple contraction cycles. The raw amplitude and frequency data were normalized to their respective values at 10 cmH_2_O and plotted as a function of pressure. For comparison, representative data from a WT Sprague-Dawley rat mesenteric lymphatic vessel are shown as previously^38^. (**D**) Confocal images of a passive lymphatic valve in open (top) and closed (bottom) states, as determined by the trans-valve pressure gradient. (**E**) Valve closure test conducted under fluorescence illumination with valve position determined by videodensitometry. With P_in_ and P_out_ equal, the valve is open; ramp-wise P_out_ elevation with P_in_ held constant, induces valve closure when the adverse pressure gradient exceeds 0.4 cmH_2_O. Scale bars: 100 µm.

We then demonstrated the functional utility of this new lymphatic-reporter rat model by characterizing their mesenteric vessel contractility. Expression of EGFP under the Prox1 promoter enabled direct measurement of the internal diameter by edge detection of the outside border of the LEC layer without impairing spontaneous contractions (Fig. 4A). The response to P_out_ elevation was typical of vessels from wild type Sprague-Dawley rats: an initial reduction in contraction amplitude that recovered over time^27^, an initial increase in contraction frequency that subsequently declined^28^, and a slight constriction^29^ (Fig. 4B). Changes in contraction amplitude and frequency as a function of pressure were nearly identical to those reported previously for wild-type rat mesenteric vessels^29^ (Fig. 4C). Likewise, the absolute contraction amplitudes and frequencies were also comparable to those of wild-type vessels^27^. The preservation of these responses is consistent with completely normal lymphatic contractile function (See Supplemental Videos). EGFP expression by the lymphatic endothelium also allowed the valve closure-pressure relationship to be determined under fluorescence illumination so that the valve was visible regardless of its orientation (Fig. 4D,E). The adverse pressure gradient required for valve closure was tested at several different baseline pressures (and therefore at different baseline diameters, not shown) and found to be consistent with the valves determined previously in wild-type rats^30^. There are several advantages to using lymphatic vessels from the Prox1-EGFP rat. First, the fluorescent edge of the LEC layer allows for direct tracking of the internal diameter by simple edge detection methods, as opposed to edge detection of outer diameter and subsequent computation of internal diameter^31^. Inner diameter is the more relevant parameter since it determines resistance to fluid flow. Second, the fluorescent lymphatic valve leaflets in the Prox1-EGFP rat allow other functional tests, such as the valve closure-pressure relationship^30^, to be determined regardless of the orientation of the valve, which becomes much more critical when performed under simple bright field illumination, where a valve can be nearly invisible when oriented 90° from the valves. Third, less cleaning of fat and connective tissue from Prox1-EGFP lymphatic vessels used for *ex vivo* studies is required for clean edge detection under fluorescence illumination, potentially allowing for better preservation of the influence(s) of adventitial cells, such as adipocytes, mast cells and/or sympathetic neurons. Finally, Prox1-EGFP rat is advantageous over the Prox1-EGFP mouse for studying the contractile properties of mesenteric lymphatics, simply because mesenteric lymphatic vessels from the mouse are non-contractile, or only very weakly contractile^29, 32, 33^, for reasons that are yet unknown, and thus do not reflect the robust contractile responses of mesenteric vessels reported in all other species studied, including humans.

**Figure 5:**
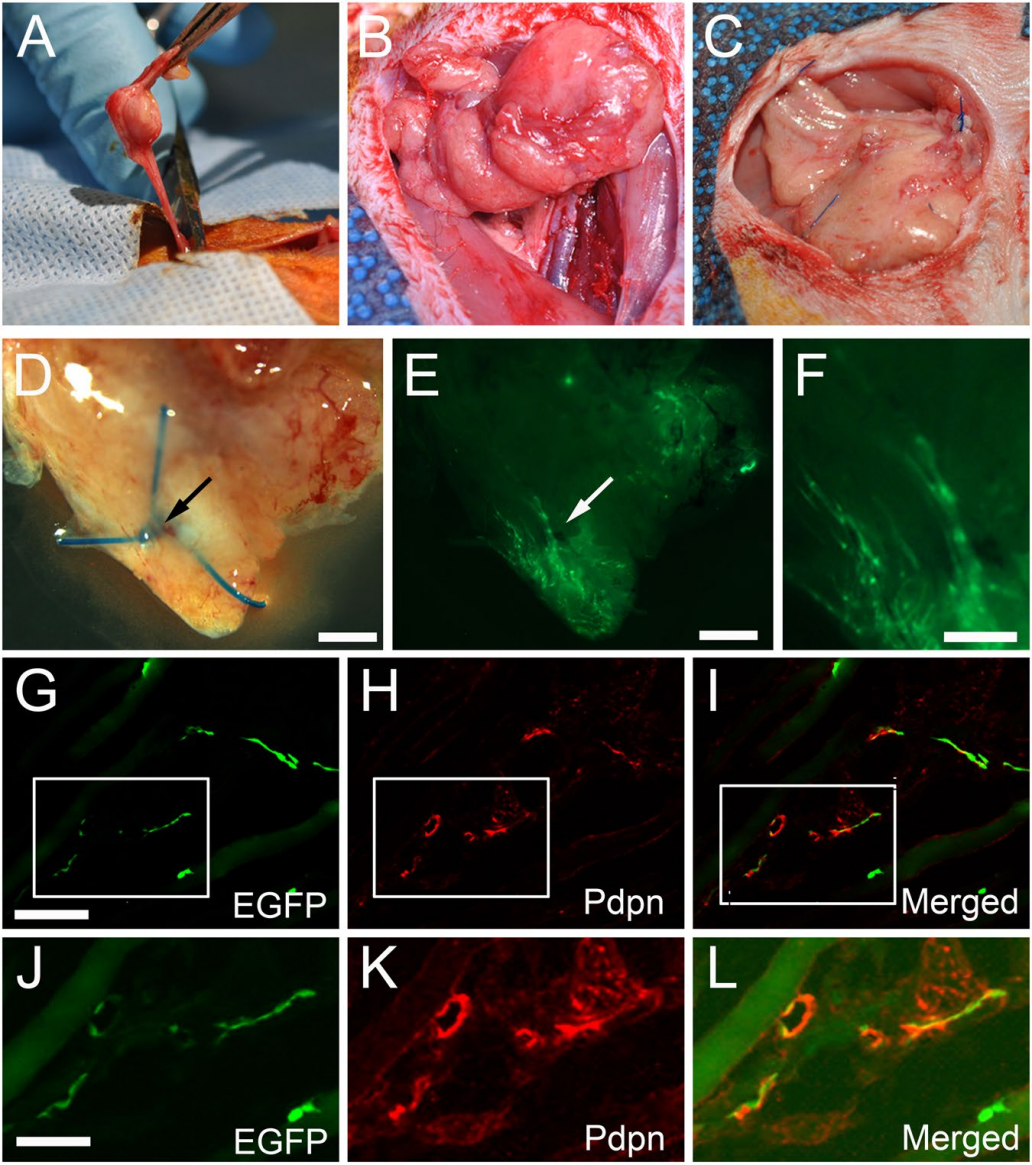
Visualization of host lymphatic contribution in vascularized lymph node transfer model. (**A**) Superficial inferior epigastric artery (SIEA) free flaps were isolated from EGFP^−^ wild type Sprague-Dawley rats on a single vascular pedicle. (**B**) SIEA flap after transfer to a Prox1-EGFP recipient rat and completion of microsurgical anastomosis of the artery and vein. (**C**) Inset of the SIEA free flap using Prolene sutures, which facilitate identification upon harvest. (**D**) Gross appearance under a stereoscope of SIEA flap harvested after 30 days. Blue suture knot (arrow) marks the host-donor boundary. (**E**) EGFP signal at the inferior pole of the transferred flap at low magnification. (**F**) Enlarged image of the edge area demonstrating lymphatic vessels with EGFP^+^ host-derived LECs. (**G–I**) Pdpn immunofluorescence assay identified host contribution to lymphangiogenesis in the donor tissue. (**J–L**) Enlarged images of boxed areas in panels (**G**–**I**), respectively. Scale bars: 1 mm (**D,E**), 0.5 mm (**F**), 100 µm (**G**), 50 µm (**J**).

Vascularized lymph node transfer (VLNT) has been shown to be an effective treatment option in select individuals with chronic lymphedema^34^. However, the mechanism behind the clinical improvement with VLNT remains uncertain and controversial. One leading hypothesis credits the spontaneous anastomoses of host and donor lymphatic vessels, while the transferred lymph nodes acts as a sump pump to drain the stalled fluid. However, this theory has been difficult to prove, largely due to the difficulty in distinguishing the donor *vs*. recipient lymphatic vessels in tissue specimens. We thus aimed to address this hypothesis by the allotransplantation of the superficial inferior epigastric artery (SIEA) flap from a wild type Sprague-Dawley rat, to a recipient Prox1-EGFP rat (Fig. 5A–C, Supplemental Fig. 2). After 30 days, the wild type transplanted flap was harvested and analyzed, and found there to have ingrowth of the recipient lymphatic vessels along the inferior pole of the flap (Fig. 5D–F). Immunofluorescence analysis of the transplanted donor flap revealed the presence of EGFP^+^ host lymphatics (Fig. 5G–L). Taken together, this study demonstrates that the Prox1-EGFP rat could be useful for investigating the effects of various surgical, microsurgical, and transplantation procedures, which have been technically challenging with Prox1-EGFP mice.

**Figure 6:**
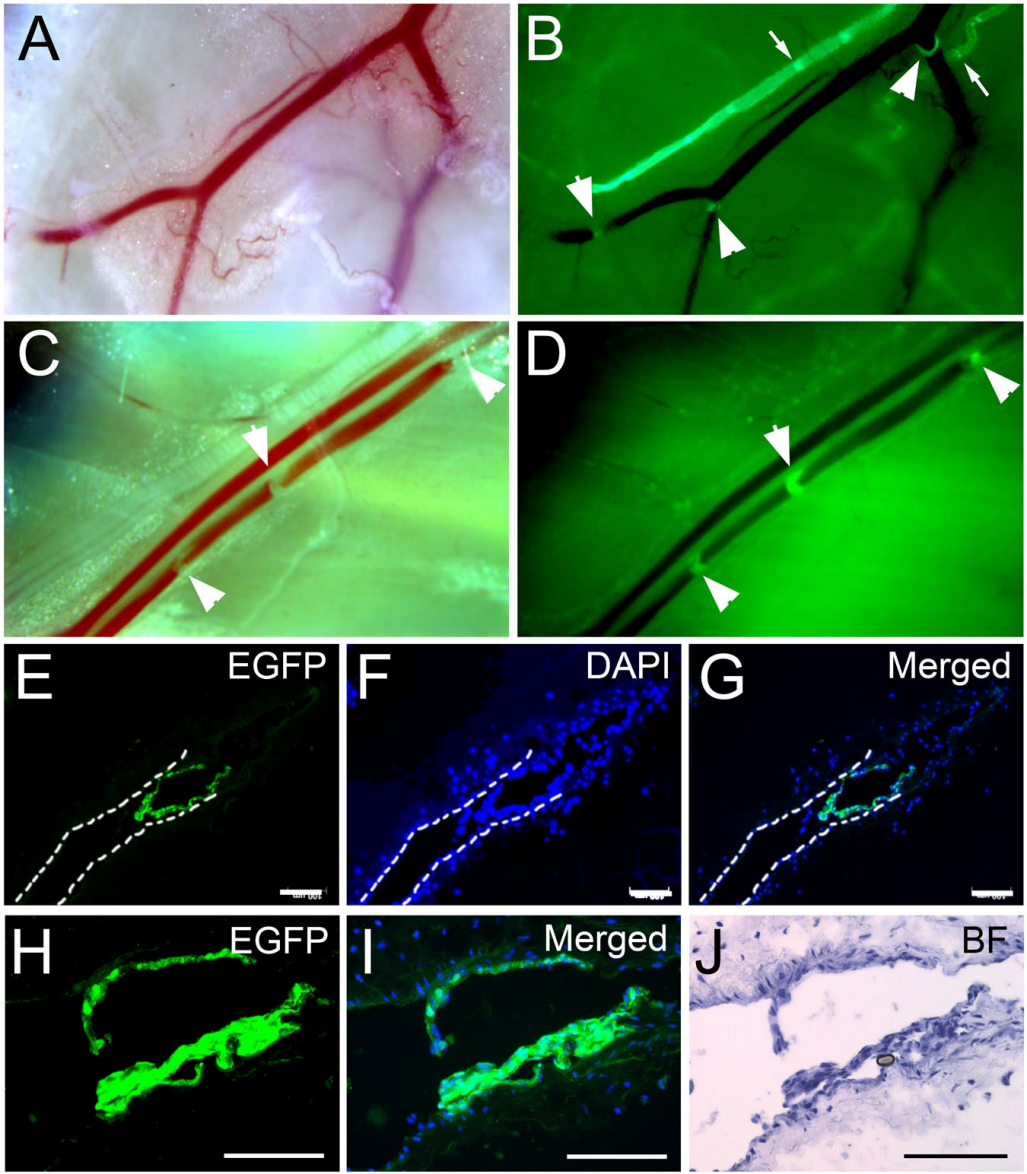
EGFP expression in venous valve endothelial cells of Prox1-EGFP rats. Comparative bright field (**A**) and fluorescent (**B**) images of arterial, venous, and lymphatic vessels of the abdominal wall in Prox1-EGFP adult mice. Blood-filled artery and vein alongside EGFP-positive lymphatic vessels are shown. Note that both venous valves (arrowheads) and lymphatic valves (arrows), along with lymphatic vessels, show strong EGFP expression. Similarly, bright field (**C**) and fluorescence (**D**) analyses revealed strong EGFP-positive valves (arrowheads) in the greater saphenous vein from Prox1-EGFP rats. (**E–G**) A cross-section of the greater saphenous vein showing robust EGFP signals in the closed valves (**E**). DAPI counter staining (**F**) and merged images (**G**) are also shown. (**H–J**) Fluorescent and bright field microscopic images showing EGFP-expressing opened venous valve (**H**), DAPI counter staining (**I**), and a corresponding hematoxylin-stained bright field image (**J**).

A previous study has elegantly demonstrated that genes regulating lymphangiogenesis also control development and maintenance of venous valve in mice^14^. In particular, both developing and mature venous valves were found to express a repertoire of lymphatic-signature proteins, including PROX1, VEGFR3, and integrin-α9, which were previously characterized as specific regulators of lymphangiogenesis^14^. Consistent with this study, we detected a strong EGFP signal in the venous valves in the abdominal layer of Prox1-EGFP mice (Fig. 6A,B). We then investigated whether Prox1 expression in venous valves seen in mice was conserved in rats using Prox1-EGFP rats. Indeed, strong expression of EGFP was detectable in the valves of the greater saphenous vein (Fig. 6C,D). Immunofluorescence analyses on cross-sectioned venous valves of Prox1-EGFP rats showed reporter positivity in the venous valve endothelial cells (Fig. 6E–G). Further histologic analyses combined with bright field imaging clearly localized the EGFP signal in the endothelial cells of valve leaflets (Fig. 6H–J). As previously reported^14^, these data support a unique identity of valve endothelial cells that is evidently different from endothelial cells in other areas of the blood vessels.

In general, rats model the metabolic, cognitive, neurological, anatomic and behavioral physiologies and pathologies of humans better than mice. Moreover, their larger size allows harvest of greater quantities of bio-specimen, such as cerebrospinal fluid, intraluminal fluid, interstitial fluid, and lymphovenous valves. Additionally, with the larger size of the rat, it is technically easier to perform sophisticated surgeries and translational investigations. Taken together, this new animal model would not only enable the convenient, direct, and possibly non-invasive visualization of lymphatic vessels and lymphoid organs, but also provide unique opportunities for the anatomic, physiological and surgical studies of the lymphatics.

## Methods

### Generation of Prox1-EGFP Transgenic Rat

All animal studies were approved by Institutional Animal Care and Use Committee (IACUC) of University of Southern California and by IACUC of the University of Missouri. All experiments were performed in accordance with relevant guidelines and regulations. The engineered mouse Prox1-harboring BAC (RP23-360I16), where the EGFP gene was inserted under the mouse Prox1 proximal promoter^9, 17^, was used to generate a Sprague-Dawley Prox1-EGFP founder line. The Prox1-harboring BAC was prepared by Cesium Chloride ultracentrifugation-based isolation method and transfected into various cell types after mixing with Lipofectamine2000 to check the EGFP expression. It was then microinjected into fertilized eggs of Sprague-Dawley rats to generate Prox1-EGFP transgenic rats in Cyagen Biosciences (Santa Clara, CA). Among 14 resulting pups, only one rat was positive for both PCR-based detection of EGFP gene and actual EGFP signals in the eyes and dermal lymphatics. This founder Prox1-EGFP transgenic rat was bred with wild type Sprague-Dawley rats to yield inbred pups and establish a transgenic line. All rats were maintained under standard conditions of room temperature and humidity, and had free access to standard diet and water ad libitum. Prox1-EGFP rats are otherwise healthy and fertile with normal litter size. Because of strong green (EGFP) signal in the eyes, transgenic rats were easily distinguishable under regular room lightings. When necessary, PCR-based genotyping was performed using primers (GATGTGCCATAAATCCCAGAGCCTAT/ GGTCGGGGTAGCGGCTGAA), yielding a ~450-bp PCR product.

### Immunofluorescence Analyses

Tissues were fixed in 4% paraformaldehyde (PFA)/phosphate buffered saline (PBS) overnight at 4 °C and embedded in CRYO-OCT compound (Sakura Finetek USA Inc.). Frozen sections (6 micron) were prepared and subjected to the standard immunofluorescence analyses^9^. The sources of the antibodies used are anti-LYVE1 antibody (AngioBio, San Diego, CA), anti-Prox1 antibody (R&D Systems, Minneapolis, MN) and anti-Pdpn antibody (PMab-2, generated by Dr. Yukinari Kato)^35^. Fluorescent images were captured under either a Keyence BZX700 digital microscope (Keyence Corp., Itasca, IL) or a ZEISS Axio Imager Z1 microscope (Zeiss, Germany).

### Flow Cytometry Analyses of Lymph Node LECs

For isolation of lymph node LECs, superficial cervical, brachial and axillary lymph nodes were removed from euthanized Prox1-EGFP rats, and incubated in DMEM containing Penicillin/Streptomycin (2,000 U/mL) at 4 °C overnight. Subsequently, they were submerged in a digestive enzyme solution (1 ml) in one well of 24-well plate, chopped into small pieces with fine surgical scissors and further incubated at 37 °C for 1 hour. The digestive enzyme solution contains dispase and collagenase (1 mg/ml, Hoffmann-La Roche, Ltd), collagenase II (50 U/mL, Worthington Biochemical, Lakewood, NJ) and DNase I (1,000 U/mL, New England Biolabs, Ipswich, MA) in PBS. LECs were then dissociated from the lymph node capsule and parenchyma by triturating the enzymatically treated lymph nodes through a 18.5 G needle and then filtered through a cell strainer (40 µm). Finally, the cells were centrifuged, harvested in culture media (EGM BulletKit, Lonza, supplemented with 20% FBS) and plated on culture plate for 12 hours. The incubated cells were washed three times using culture media to remove unattached and/or dead cells. Notably, 60~80% cells among the attached population were GFP-positive, suggesting that the majority of attached cells were lymph node LECs and that most other cell types were eliminated. The remaining cells were then trypsinized, fixed with 1% PFA for 10 minutes at 4 °C, washed 3 times with cold PBS, and attached with anti-Lyve1 antibody (11–034, AngioBio) or non-specific rabbit IgG (as a negative control, Sigma-Aldrich) overnight at 4 °C. Next day, the cells were washed 3 times with cold PBS, incubated with donkey anti-rabbit IgG antibody conjugated with Alexa Fluor 647 (A-31573, Invitrogen) for 1 hour at 4 °C, followed by 3 wishes with cold PBS, and then subjected to flow cytometer analyses (BD LSR II Flow Cytometer). We used forward scatter (FSC) and side scatter (SSC) to find viable, single cell events and eliminate any debris, dead cells and clumps, and set the threshold for GFP and Lyve1 signals based on unstained wild type lymph node cells. Normal IgG-stained cells and Lyve1-stained cells were then analyzed and plotted to generate bivariate histograms (Fig. 3M). Consistent with our observation that the GFP-positive cells comprised of most of the attached cells in the initial culture plate, Lyve1^+^GFP^+^ cells made up for ~74% of all gated events.

### Study of Mesenteric Lymphatic Contractility

An arcade of mesentery was excised, placed in 4 °C Krebs buffer and shipped overnight to the University of Missouri. Viability was not impaired if vessels were studied within 36 hours^30^. Upon arrival, collecting lymphatics were dissected from the mesenteric fat, cleaned, cannulated on micropipettes, and studied under controlled pressure conditions at 37 °C^27^. Contractile responses were assessed in standard Krebs buffer, first under bright field illumination and then under fluorescence illumination. The latter protocols used an Olympus IX81 microscope equipped with a Yokagawa spinning disk, using 488 nm laser excitation and emission detection at 530 nm emission; images were collected in Metamorph using a Hamamatsu Flash 4.0 camera (30 fps). Diameter was measured by edge detection Valve closure tests were performed as described previously^36^ after 20 min equilibration in calcium-free Krebs to block contractions; under those conditions valve position was determined exclusively by external adjustment of the input (P_in_) and output (P_out_) pressures.

### Vascularized Lymph Node Transfer

Free flaps consist of transplantable tissue segments whose blood supply is severed at the original site, as illustrated Supplemental Fig. 2. Free flaps containing the lymph nodes and surrounding subcutaneous tissue supplied by the superficial inferior epigastric artery (SIEA) were isolated and excised from 6 wild type Sprague-Dawley rats and transplanted to 6 Prox1-EGFP reporter rats using a Leica M535 F50 surgical microscope (Leica Micro., Wetzlar, DE). Flap elevation, transplant, and microvascular anastomosis was performed as previously described^37^. A 2 cm transverse skin incision was made 1 cm superior to the inguinal crease in Sprague-Dawley rats. The subcutaneous tissue was visualized using an inferior incision followed by marginal dissection in order to expose all the tissue for harvest and transplantation. The flap, measuring approximately 4 cm^2^, was then isolated and elevated to expose the vascular pedicle. The superficial inferior epigastric artery and vein (SIEA and SIEV) were visualized and dissected to their origin from the common femoral artery and vein. The deep femoral artery and vein were ligated with chemical cautery and the superficial femoral artery and vein distal to the branch point of the SIEA and SIEV was tied using 6–0 Prolene sutures and cut in order to elevate the flap. Next, the superficial femoral artery and vein were severed with microsurgical scissors proximal to the branch point of the SIEA and SIEV. A similar flap dissection and elevation was performed in the Prox1-EGFP reporter rat. The flap was then harvested by ligating the SIEA and SIEV for histological analysis. After cauterizing the profunda femoris artery and vein and ligating the superficial femoral artery and vein, the flap from Sprague Dawley rat was placed in the surgical field. An arterial and venous end-to-end anastomosis was then performed using 10–0 Nylon (Ethicon Inc., Cincinnati, OH) sutures and patency was verified. The margins of the 4 cm^2^ flap were then sutured to the subcutaneous tissue with 5–0 Prolene for later identification and the incision closed with skin staples. At post-operative day 30, the transplanted donor tissues were harvested for photographic and histological analysis.

## Electronic supplementary material

Supplemental Information

Video1

Video2

Video3
