# Supplementary material for: Development and Characterization of A Novel Prox1-EGFP Lymphatic and Schlemm’s Canal Reporter Rat
Source: Sci Rep. 2017 Jul 17;7:5577. doi: 10.1038/s41598-017-06031-3 (PMC5514086; doi:10.1038/s41598-017-06031-3)
Supplement: Supplementary file 1 — Supplemental Information [file 41598_2017_6031_MOESM1_ESM.pdf]

SUPPLEMENTAL INFORMATION

Development and Characterization of A Novel Prox1-EGFP Lymphatic and Schlemm's canal Reporter Rat

Eunson Jung [1,2], Daniel Gardner [1], Dongwon Choi [1,2], Eunkyung Park [1,2], Young Jin Seong[1,2], Sara Yang [1,2], Jorge Castorena-Gonzalez [3], Antoine Louveau [4], Zhao Zhou [1], Gene K. Lee [1], David P. Perrault [1], Sunju Lee [1,2], Maxwell Johnson[1], George Daghlian [1,2], Maria Lee [1,2], Yeo Jin Hong [1,2], Yukinari Kato [5], Jonathan Kipnis [4], Michael J. Davis [3], Alex K. Wong [1], and Young-Kwon Hong [1,2]

[1] Department of Surgery, [2] Department of Biochemistry and Molecular Biology, Norris Comprehensive Cancer Center, Keck School of Medicine, University of Southern California, Los Angeles, California; [3] Department of Medical Pharmacology and Physiology, University of Missouri, Columbia, Missouri; [4] Center for Brain Immunology and Glia, Department of Neuroscience, University of Virginia School of Medicine, Charlottesville, Virginia; [5] Department of Regional Innovation, Tohoku University Graduate School of Medicine, Sendai, Japan.

Eunson Jung, Daniel Gardner and Dongwon Choi contributed equally to this study.

Correspondence should be addressed to:

Alex K. Wong, M.D., F.A.C.S.
Division of Plastic and Reconstructive Surgery
Department of Surgery
Keck School of Medicine of USC
1510 San Pablo Street, Suite 415
Los Angeles, CA 90033-4680
Tel. (323) 442-7920
Fax. (323) 442-7573
E-mail: Alex.Wong@med.usc.edu

Young-Kwon Hong, Ph.D.
Department of Surgery
Department of Biochemistry and Molecular Biology
University of Southern California
Norris Comprehensive Cancer Center
1450 Biggy St. NRT6501
Los Angeles, CA 90033
Tel: 323-442-7825
FAX: 323-442-7844
E-mail: young.hong@usc.edu

SUPPLEMENTAL FIGURE

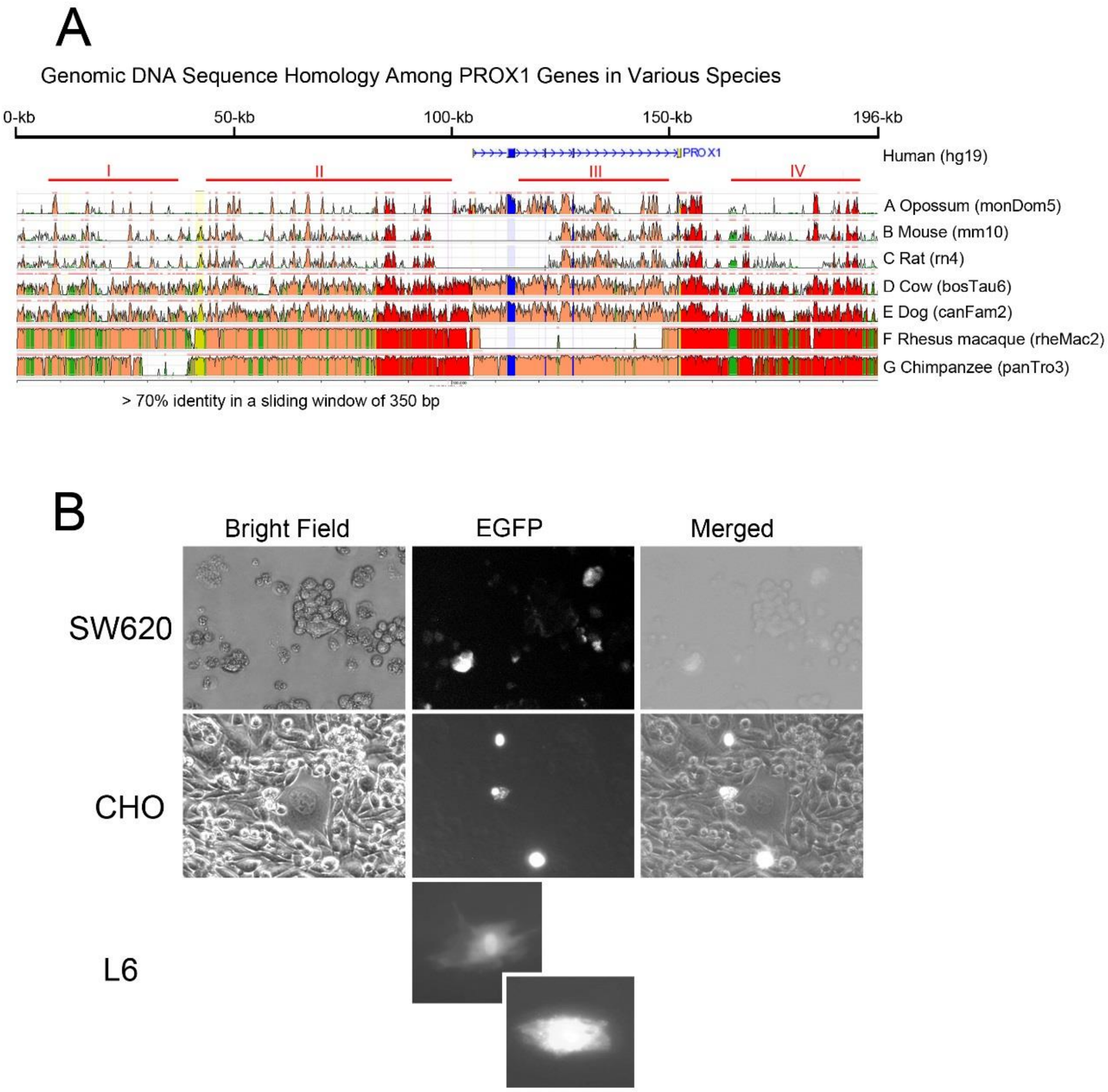

Supplemental Figure 1. Cross species conservation of the genomic sequence and function of the engineered mouse Prox1-harboring BAC. (A) Multi-species alignments of genomic DNA sequences corresponding to the genomic region contained in the mouse Prox1 BAC clone (RP23-360I16). The genomic sequences from opossum, mouse, rat, cow, dog, Rhesus macaque and chimpanzee were aligned against the corresponding human sequences using the Evolutionary Conservation of Genomes (ECR) browser. Genome reference versions are shown in parenthesis. Clustered conserved areas are shown (I, II, III and IV). (B) Functional test of the Prox1-EGFP BAC in non-mouse cells. The mouse Prox1 BAC clone was transfected into Prox1-expressing non-mouse cells, such as SW620 (human colon carcinoma), CHO (Chinese hamster ovary cells), and L6 (rat myoblasts). The mouse Prox1 BAC clone indeed expressed a strong GFP signal in the three non-mouse cells.

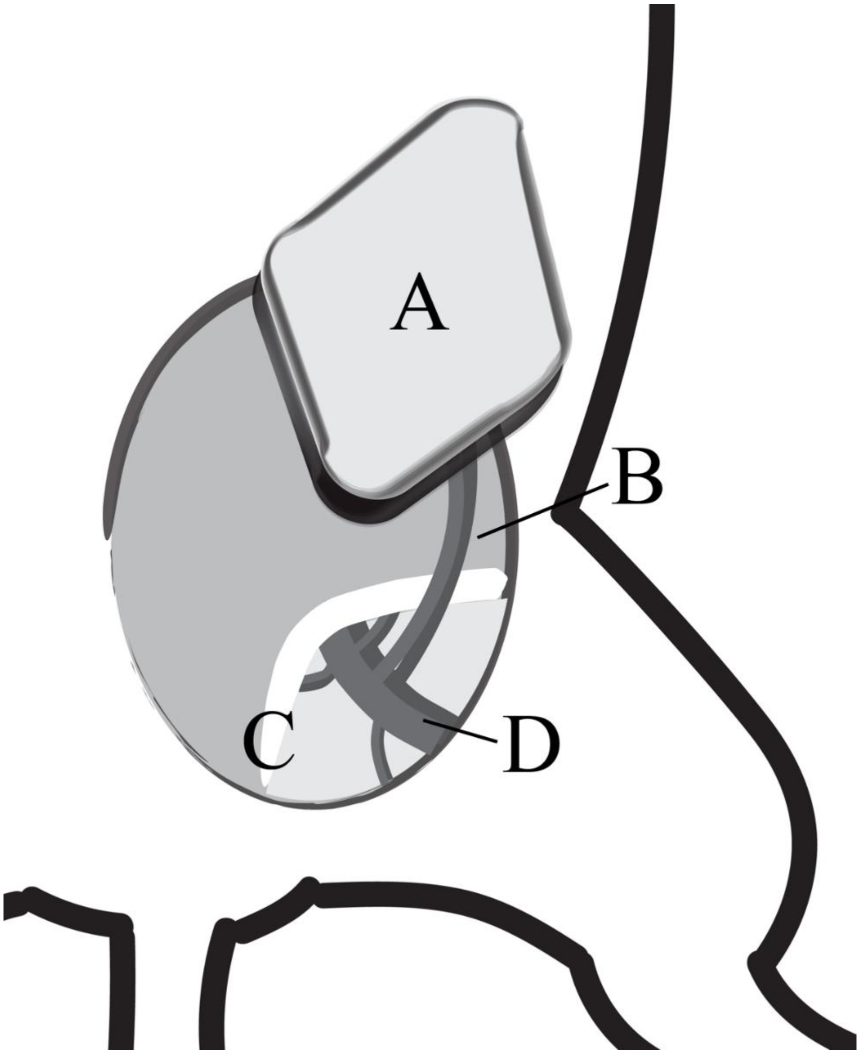

Supplemental Figure.2 In the donor animal, the fat pad (A, groin flap) was identified and isolated from the surrounding tissue. The superficial epigastric vessels (B) were then visualized and isolated. The femoral vessels were ligated distal to the inguinal ligament (C) and proximal to the branch point of the superficial epigastric vessels. The femoral vessels (D) distal to the superficial epigastric vessels were tied with suture, cut, and would be later anastomosed to the femoral vessels in the recipient animal. In the recipient animal, the groin flap was isolated and resected in a similar way. Once the groin flap in the recipient animal had been removed, the groin flap from the donor animal was set in place and the donor femoral vessels were anastomosed in an end-to-end fashion to the cranially located recipient femoral vessels.

Information for Supplemental Videos

Data collected on Prox1GFP rat mesenteric lymphatics 2-9-16 by Mike Davis and Jorge Castorena-Gonzalez.

Vessels were shipped at 4oC overnight, dissected and cannulated on 80 um pipettes, pressurized at both ends as shown below.

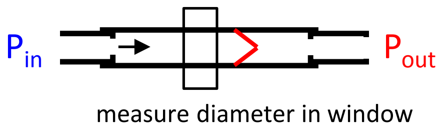

Brightfield image

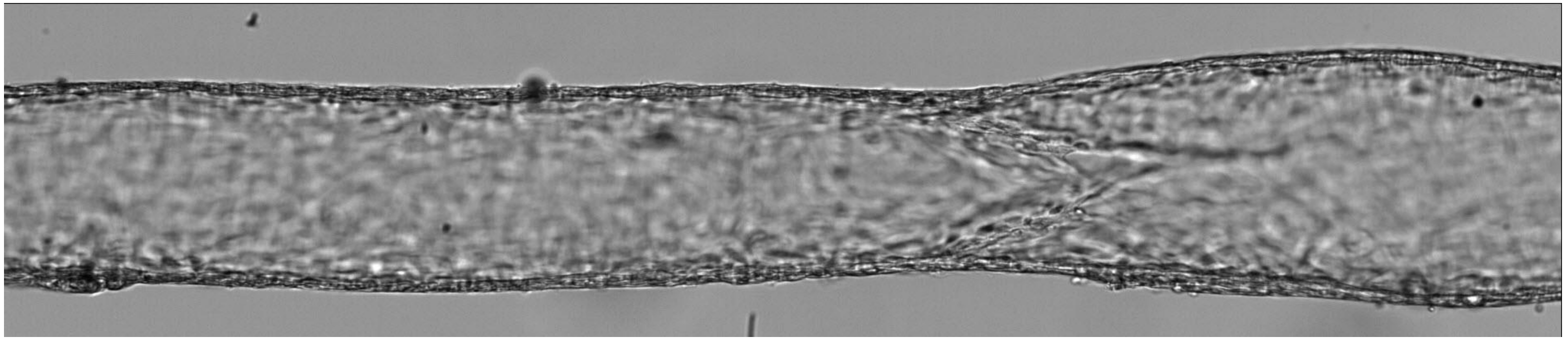

Confocal stack reconstruction

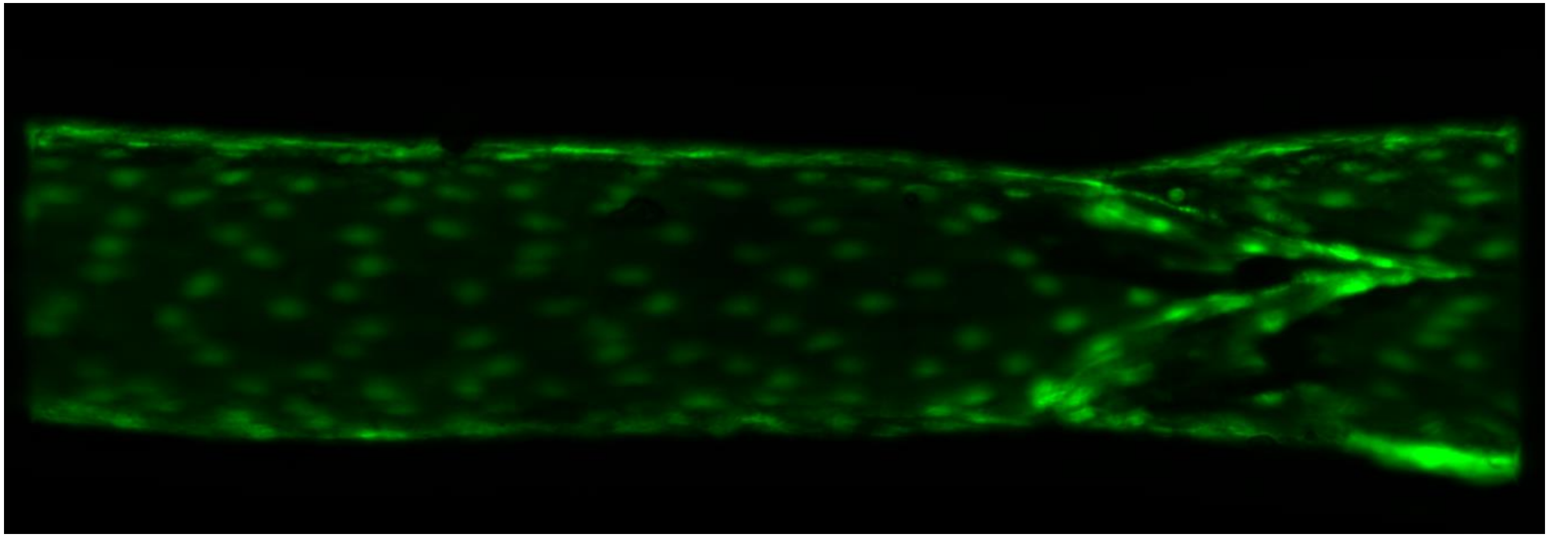

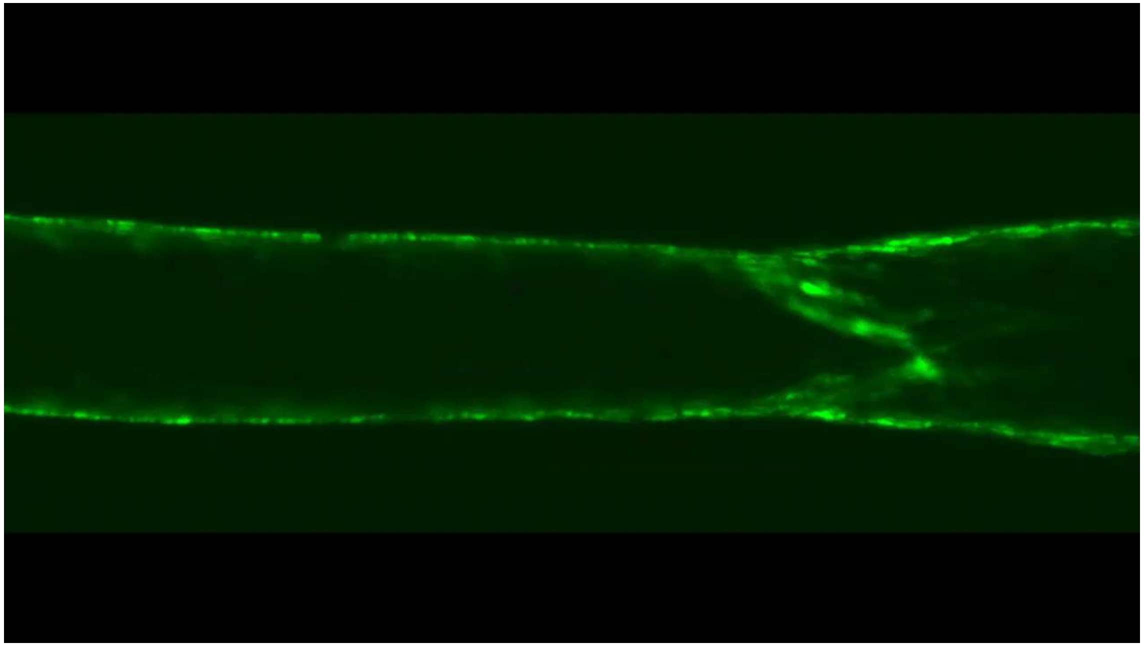

Please watch
Supplemental Video1

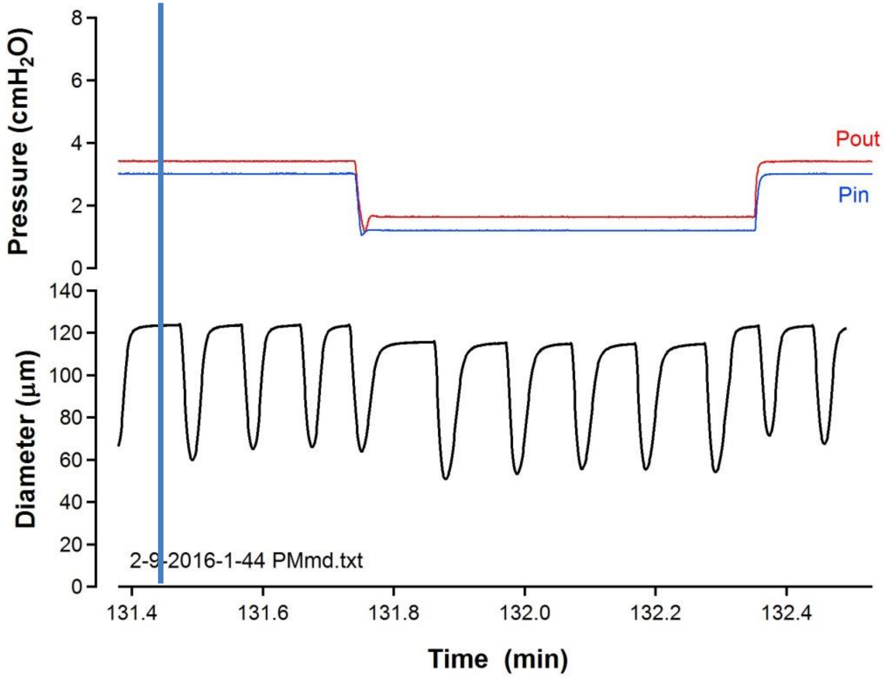

Diameter is measured on the input side of the valve before and during a pressure step. The pressures are set so they are not exactly equal in order to show how the valve opens/closes during contractions.

Normally, outer diameter is measured by edge detection in brightfield images;
In the Prox1gfp vessel, internal diameter can be directly
measured by edge detection.

Valve closure
Test at low
pressure

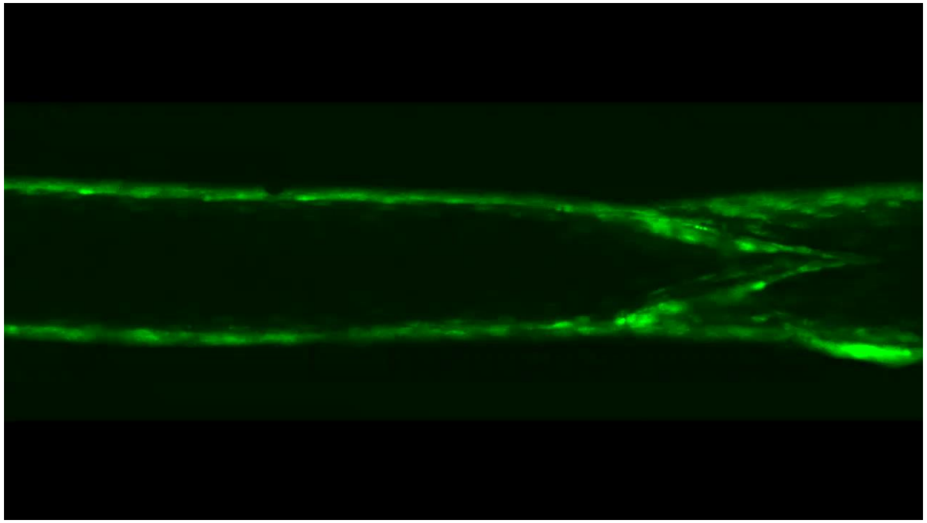

Please watch
Supplemental Video2

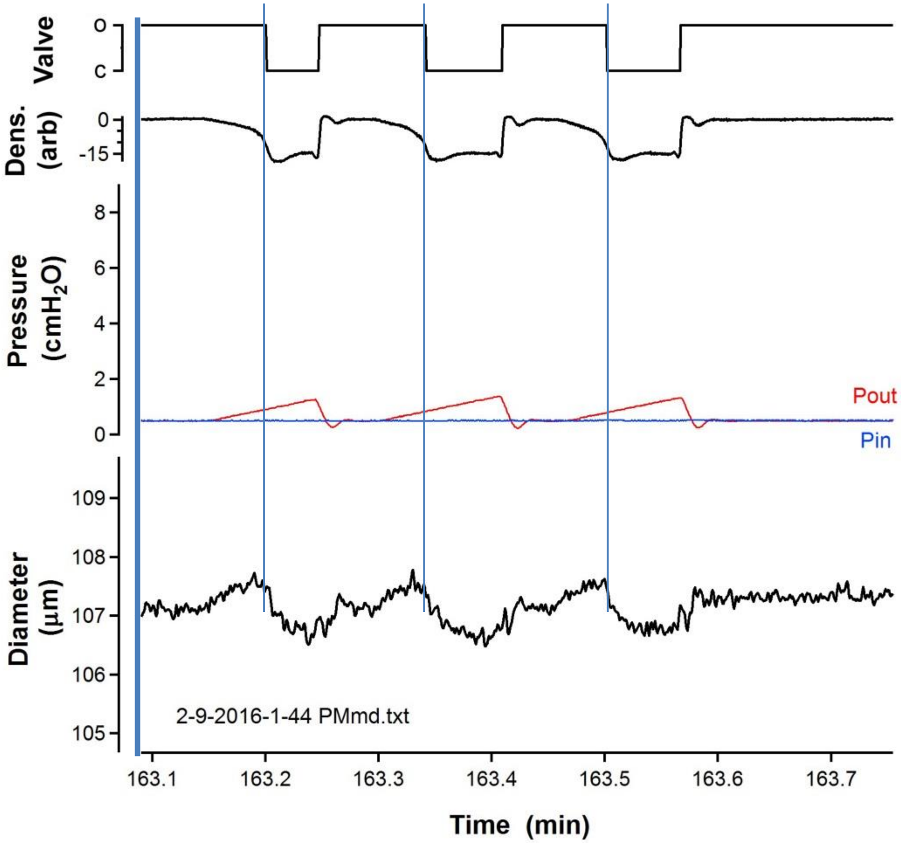

Output pressure (red trace)
is raised slowly until the valve closes.
Valve position is detected by a
densitometer widow placed between
the leaflets.

Test is repeated 3x. The vertical lines
show the points in time when the valve
closes.

The pressure gradient required to
close the valve is the difference between
The red and blue lines at the point of closure.

Valve closure Test at high pressure

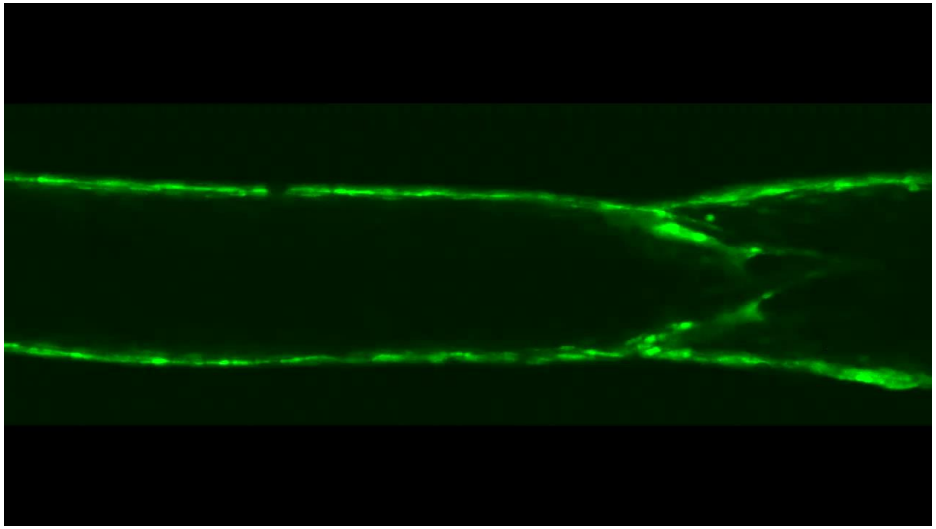

Please watch Supplemental Video3

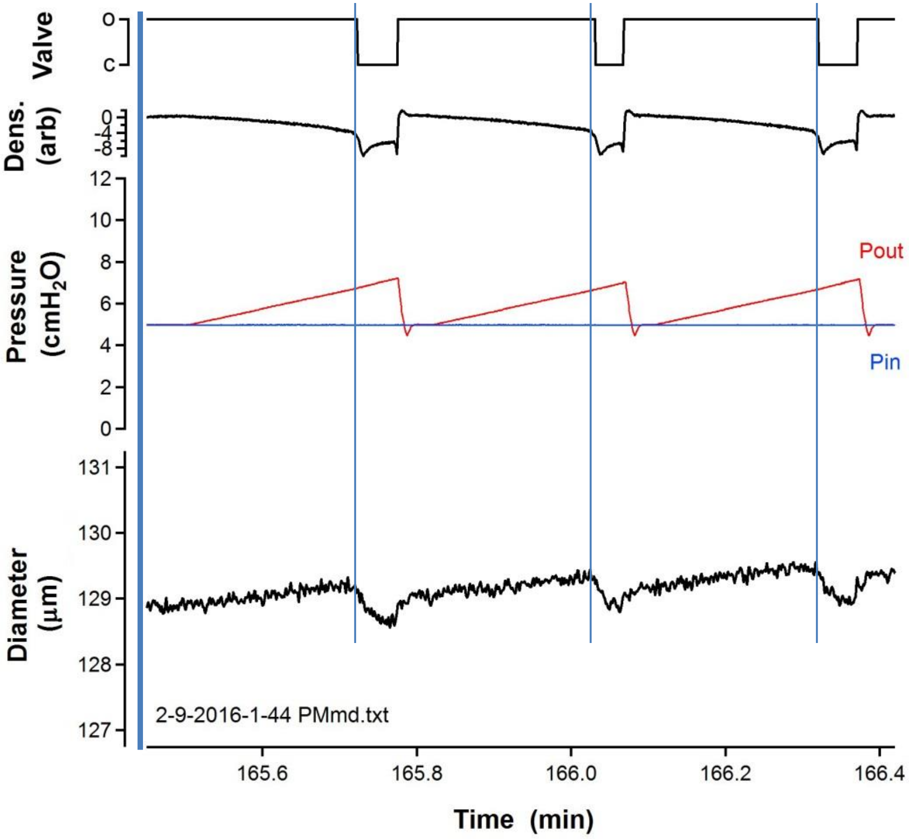

Same test as in previous slide except the vessel is expanded by a higher baseline pressure at the start of the test. There is more tension on the valve leaflets so a higher pressure gradient is required for closure.
